# Is nurses’ clinical competence associated with their compassion satisfaction, burnout and secondary traumatic stress? A cross‐sectional study

**DOI:** 10.1002/nop2.636

**Published:** 2020-10-04

**Authors:** Mohammad Ali Zakeri, Gholamreza Bazmandegan, Hamid Ganjeh, Maryam Zakeri, Sekineh Mollaahmadi, Ali Anbariyan, Zahra Kamiab

**Affiliations:** ^1^ Non‐Communicable Diseases Research Center Rafsanjan University of Medical Sciences Rafsanjan Iran; ^2^ Clinical Research Development Unit, Ali‐Ibn Abi‐Talib Hospital Rafsanjan University of Medical Sciences Rafsanjan Iran; ^3^ Department of Family Medicine, Ali‐Ibn Abi‐Talib Hospital, School of Medicine Rafsanjan University of Medical Sciences Rafsanjan Iran; ^4^ Physiology‐Pharmacology Research Center, Research Institute of Basic Medical Sciences Rafsanjan University of Medical Sciences Rafsanjan Iran

**Keywords:** burnout, clinical competence, compassion satisfaction, nurse, professional quality of life, secondary traumatic stress

## Abstract

**Aim:**

The current study aimed to investigate the relationship between clinical competence and subscale of professional quality of life (ProQOL).

**Methods:**

This cross‐sectional study was conducted from November 2018 to May 2019 on 291 nurses working in public hospitals in south Iran (Rafsanjan). The nurses were selected by convenience sampling method. Data were collected using the ProQOL questionnaire that included three subscales: compassion satisfaction, secondary traumatic stress and burnout and the Competency Inventory for Registered Nurse (CIRN) that measured clinical competence.

**Results:**

A significant positive relationship was observed between compassion satisfaction and clinical competence. A significant negative association was found between compassion satisfaction and secondary traumatic stress/burnout and also between secondary traumatic stress and clinical competence. The results of regression analysis indicated that compassion satisfaction was the best predictor of secondary traumatic stress (*R* ^2^ = 65%), burnout (*R* ^2^ = 40%) and clinical competence (*R* ^2^ = 12%). Moreover, secondary traumatic stress was found to be the best predictor of compassion satisfaction (*R* ^2^ = 53%).

## 1 INTRODUCTION

At present, the provision of quality and safe care is a primary goal of health care worldwide (Numminen et al., 2015). Nurses, the key members of the treatment team, are often the first ones who are in contact with patients and their families (Singh & Hubbard, 2011). With the advancement of technology, clinical settings have rapidly changed and become more sophisticated; nurses then must be able to focus on and cope with these changing clinical situations by creating and using resources and carrying out appropriate activities (O'shea, 2003). The description and development of competencies are a major challenge for nurses who manage patient care (Furukawa et al., 2010).

### 1.1 Background

Nurses' clinical competence is one of the essential requirements for providing safe and effective caregiving (Kim et al., 2015) and is one of the main elements in providing nursing care based on professional performance standards (Bahreini et al., 2010). Nurses need high‐level clinical expertise to accurately assess the patient's condition and deal with problems that may occur during treatment (Park & Kim, 2009).

Aued et al. (2016) showed that identifying clinical competence and nurses’ access to this competence were important achievements for managing individuals. Nurse managers can use them to allocate nurses better and provide opportunities for their professional development (Aued et al., 2016). The recognition of various factors affecting the development of clinical competence is very important (Bench et al., 2003). Assessing and improving clinical competence is an ongoing and dynamic process and many factors can affect the level of competence of nurses (Yanhua & Watson, 2011). A study by Mirlashari et al. on nurses in the intensive care unit (ICU) found that factors such as marital status, employment status and interest in working in the ICU should be taken into consideration for inducing the highest level of clinical competence in nurses (Mirlashari et al., 2016).

A study by Memarian et al. (2007) showed that factors such as effective communication, career interest, accountability work experience and personal factors such as ethical and professional principles, knowledge and skills affect the competence of nurses. Professional factors such as effective management, control and monitoring and external environmental factors such as an efficient education system and providing technology are other factors affecting the competence of nurses. Yanhua and Watson (2011) review showed that there is a growing concern about the competence of nurses, which has led nursing managers to pay special attention to it and related factors.

Nurses’ clinical competence must be assessed regularly because of the ongoing changes in therapeutic settings. However, nurses may have a low quality of life, which can affect their clinical competence (Kim et al., 2015). Recently, the focus has been shifted on concepts related to the professional quality of life (ProQOL) and job performance of nurses, including their clinical function and competence (Vagharseyyedin et al., 2011). Nurses are exposed to occupational stress and they experience the highest levels of stress in the workplace (Sonke et al., 2015).

Nayomi’s (2016) review revealed emergency cases, high workload, understaffing, lack of support or positive feedback from senior nursing staff, poor working relationship, demanding communication and relationships with patients and relatives have been the main sources of stress for nurses.

Thus, workplace stress has caused job dissatisfaction and burnout for nurses (Commission, 2012). Given that nurses are the main health providers who observe pain and suffering (Farhadi et al., 2013), job‐related stress responses such as compassion fatigue and occupational burnout have a negative impact on the nurses’ ProQOL, clinical decision‐making and patient care quality (Thomas, 2012) and hence, affecting the clinical competence of nurses.

The term "professional quality of life" or ProQOL with three components of compassion satisfaction, secondary traumatic stress and occupational burnout, refers to the positive and negative emotions that a person describes in his/her job as a contributor and a rescuer (Stamm, 2002; Stamm, 2010). Compassion satisfaction occurs when altruistic empathy behaviours are used to reduce a patient's suffering and cause the caregiver to deal with the negative aspects of his or her work life (Sacco & Copel, 2018). Burnout is a negative alternative to the patient's suffering, which can be seen in the achievements of patient care (Hinderer et al., 2014).

ProQOL is an important concept for the health system and healthcare providers and is associated with personality traits and the occupational environment (Pashib et al., 2016). Kim and Choi found that over 50% of emergency nurses had a poor quality of life at work and low ProQOL (Kim & Choi, 2012). It has been shown that employees with higher ProQOL, organizational identity, higher satisfaction and job performance were less willing to leave a job (Lee et al., 2007). In particular, nursing practice is a compassion‐based satisfaction experience, which can have a negative impact on nurses’ physical and mental health and care performance (Kim et al., 2015). Satisfaction with compassion and related avoidance behaviours can prevent nurses’ empathy for patients and ultimately reduce their competence and quality of clinical practice (Boyle, 2011; Kim & Choi, 2012).

Given that the goal of nursing training is to work in complex and unpredictable clinical settings, special attention should be paid to the complicated nature of the competency and the characteristics required for it (Levett‐Jones et al., 2011). Nonetheless, few studies have examined the effect of ProQOL on the clinical competence of nurses. On the other hand, studies have identified factors influencing nurses' clinical performance and competence such as job stress, job satisfaction and demographic characteristics (Boyas et al., 2012). Since the ProQOL is also related to job performance, this study aimed to determine the relationship between clinical competence and the ProQOL dimensions (compassion satisfaction, burnout and secondary traumatic stress) in Iranian nurses and provide implications for improving their ProQOL and clinical competence. Specific objectives of this study were to identify: (a) the level of clinical competence; (b) assess the three variables of compassion satisfaction, burnout and secondary traumatic stress; (c) the relationship between demographic characteristics, ProQOL and clinical competence; (d) the relationship between ProQOL and clinical competence and; and (f) the regression between variables of ProQOL and clinical competence.

## 2 METHOD

### 2.1 Sample size and sampling

This cross‐sectional study was conducted on all 340 nurses working in public hospitals in southern Iran (Ali‐Ibn Abi‐Talib Hospital and Moradi Hospital of Rafsanjan). The nurses were selected by the convenience sampling method. The inclusion criteria were as follows: nurses working in the healthcare sector that provide direct care for patients; with at least one year of experience; and no management position and the willingness to participate in the study. The exclusion criteria were as follows: the respondents who were not nurses; pregnant or breastfeeding nurses; and those with a history of mental illness and those who did not complete the questionnaire (even one question left unanswered).

### 2.2 Data Collection

A total of 300 nurses working in the two hospitals who met the inclusion criteria entered the study by the census method. Three hundred questionnaires were distributed over six months (November 2018 – May 2019) among the nurses and they were asked to return the questionnaires after completing them and 291 copies were returned (response rate: 97%).

### 2.3 Measurements

#### 2.3.1 Socio‐demographics

A socio‐demographic information questionnaire was also completed by the respondents. The questionnaire assessed the respondents’ age, gender, level of education, marital status, number of children, nursing service records, work shift, income level, working hours per month, overtime, the number of overtime hours per month, a second job (if any), the department, job satisfaction and satisfaction with colleagues, absenteeism, adaptation of the programme to the person's tendency, diseases or specific problems and a history of drug use.

#### 2.3.2 Competency Inventory for Registered Nurses

The Competency Inventory for Registered Nurses (CIRN) was developed in the mainland China for assessing the clinical competency of nurses in different clinical settings. This 55‐item questionnaire was validated by Liu et al. (2009) in Macao, China. It consists of seven aspects of nursing competence: clinical care (10 items), leadership (9 items), interpersonal relationships (8 items), ethical/legal performance (8 items), professional development (6 items), coaching/training (6 items) and the desire for research/critical thinking (8 items). The internal consistency of the questionnaire with Cronbach's alpha coefficient was 0.91, ranging from 0.72–0.90 (Liu et al., 2009).

This questionnaire was translated into Persian by Ghasemi et al. (2014) for use in Iran and its reliability (internal consistency) was confirmed with a total Cronbach's alpha coefficient of 0.87 for the whole questionnaire (with the corresponding values of the subscales ranging from 0.88–0.97). The items in this questionnaire are scored on the five‐point Likert scale ranging from 0–4 (0 = lack of competence, 1 = low competence, 2 = limited competence, 3 = sufficient competence and 4 = very high competence). The total score varies from 0–220 points and the higher the total score, the higher the competences (scores ranging from 165–220 show high competence, scores ranging from 110–165 show moderate competence and scores less than 110 show low competence). In the present study, Cronbach's alpha coefficient for the whole questionnaire was 0.93. Moreover, the reliability of the nursing competence tool was 0.97 using Cronbach's alpha coefficient. Cronbach's alpha coefficients for clinical care, leadership, interpersonal relationships, ethical/legal performance, professional development, coaching/training and the desire for research/critical thinking were 0.88, 0.86, 0.85, 0.82, 0.84, 0.83 and 0.84, respectively.

#### 2.3.3 The ProQOL Scale, version 5

Pashib et al. (2016) translated the ProQOL questionnaire for use in Iran and assessed its validity and reliability. The questionnaire consists of three subscales as follows: compassion satisfaction, secondary traumatic stress and burnout. This questionnaire contains 30 items scored on a 5‐point Likert scale (from never = 1 to always = 5). Each subscale consists of 10 items with an independent score that is calculated as the sum of the relevant items. In addition, in some studies, the concept of compassion fatigue has been conceptualized through burnout and secondary traumatic stress, which refers to the negative effects of care (Stamm, 2002; Stamm, 2010).

High scores of compassion satisfaction indicate one's satisfaction with his/her ability in providing services and care and high scores of burnout and secondary traumatic stress indicate that the person is at a high risk of compassion fatigue. Furthermore, high scores on secondary traumatic stress indicate that a person is vulnerable to stress symptoms such as hopelessness and discomfort. In all three domains, the standard scoring is based on high (above 42), moderate (23–41) and low scores (below 22). The validity of the ProQOL questionnaire was assessed via the content validation method and its reliability was established through Cronbach's alpha coefficient in Pashib et al.’s study. The Cronbach's alpha coefficients for compassion satisfaction, compassion fatigue and burnout were 0.82, 0.8 and 0.47, respectively (Pashib et al., 2016). The answers to items (1, 4, 15, 17 and 29) in the secondary traumatic stress scale must be scored inversely. In the present study, the reliability of the ProQOL scale was 0.80 using Cronbach's alpha coefficient and the corresponding values for compassion satisfaction, secondary traumatic stress and burnout were 0.87, 0.76 and 0.57, respectively.

### 2.4 Statistical analysis

Descriptive statistics (means, standard deviation, minimum and maximum scores) were used to analyse the participants’ demographic data. Besides, bivariate correlations and regression analyses were used to examine the relationships between the variables (CI = 95%, *p* = .05). All statistical procedures were performed using ibm spss22.

### 2.5 Ethical Considerations

This study was conducted after obtaining the code of ethics (IR.RUMS.REC.1397.213) from the Ethics Committee of Rafsanjan University of Medical Sciences. The nurses were asked to complete the questionnaire after obtaining written consent and explaining the goals and importance of the study. To comply with ethical requirements, the participation was voluntary and the participants’ information was kept confidential and was used only for the purpose of this study.

## 3 RESULTS

### 3.1 Participants

The sample in this study consisted of 291 nurses with a mean age of 33.24 (*SD* 6.2) years. Table 1 shows the baseline characteristics of the participants. Most participants were female (240, 82.4%), married (243, 83.5%), Bachelor of Nursing (264, 90.7%) and working in the ICU ward (60, 20.6%) and had 5–10 years of work experience (134, 46.4%). Most participants were satisfied with their jobs and colleagues (203, 69.8%). Rotating shifts had the highest frequency (262, 90%).

**Table 1:** Demographic variables of participants (*N* = 291)

Participant characteristics	*N* (%)
Gender	
Female	240 (82.4)
Male	51 (17.6)
Marital status	
Unmarried	43 (14.8)
Married	243 (83.5)
Divorced	4 (1.4)
Widowed	1 (0.3)
Children	
Yes	176 (60.5)
No	115 (39.5)
Academic level	
Bachelor of nursing	264 (90.7)
Master of nursing	27 (9.3)
Job experience (year)	
>5	81 (27.8)
5–10	134 (46.4)
11–15	40 (13.7)
16–20	23 (7.9)
<20	13 (4.2)
Satisfaction with nursing job	
Yes	203 (69.8)
No	88 (30.2)
Taking medication	
Yes	31 (10.7)
No	260 (89.3)
Satisfaction with colleagues	
Yes	261 (89.7)
No	30 (10.3)
Shift	
Morning	12 (4.1)
Evening	6 (2.1)
Night	11 (3.8)
Rotational	262 (90.0)
Hospital wards	
CCU	19 (6.5)
ICU	60 (20.6)
Emergency	50 (17.2)
Paediatric	18 (6.2)
Neonatal and NICU	22 (7.6)
Maternity	33 (11.3)
Medical	17 (5.8)
Neurology	12 (4.1)
Surgery	26 (8.9)
Oncology	6 (2.1)
Endocrine	10 (3.4)
ENT	9 (3.1)
Psychiatry	9 (3.1)

Note

Data were presented numerically (%). The sample consisted of 291 nurses with the mean age 33.24 ± 6.2 years.

Abbreviations: CCU, coronary care unit; ENT, ear, nose and throat; ICU, intensive care unit; NICU, neonatal intensive care unit.

### 3.2 Descriptive statistics

Table 2 shows the distribution of the participants’ scores on the ProQOL scales. The highest mean value was related to the compassion satisfaction (38.89 *SD* 6.49) and the lowest mean value was found in secondary traumatic stress (26.02 *SD* 5.66).

**Table 2:** Distribution of ProQOL variables (*N* = 291)

Variable	Mean	*SD*	Min	Max	Skew	Kurtosis
Compassion satisfaction (ProQOL)	38.89	6.492	18	50	−0.682	0.263
Compassion fatigue (secondary Traumatic stress and burnout)	47.84	10.040	26	78	0.210	−0.360
Secondary traumatic stress (ProQOL)	26.02	5.669	14	46	0.525	0.392
Burnout (ProQOL)	21.84	5.658	10	39	0.120	−0.624

Note

Data were presented as mean, *SD*, minimum, maximum, skewness and kurtosis for each variable.

Abbreviation: ProQOL, Professional quality of life.

**Figure 1:**
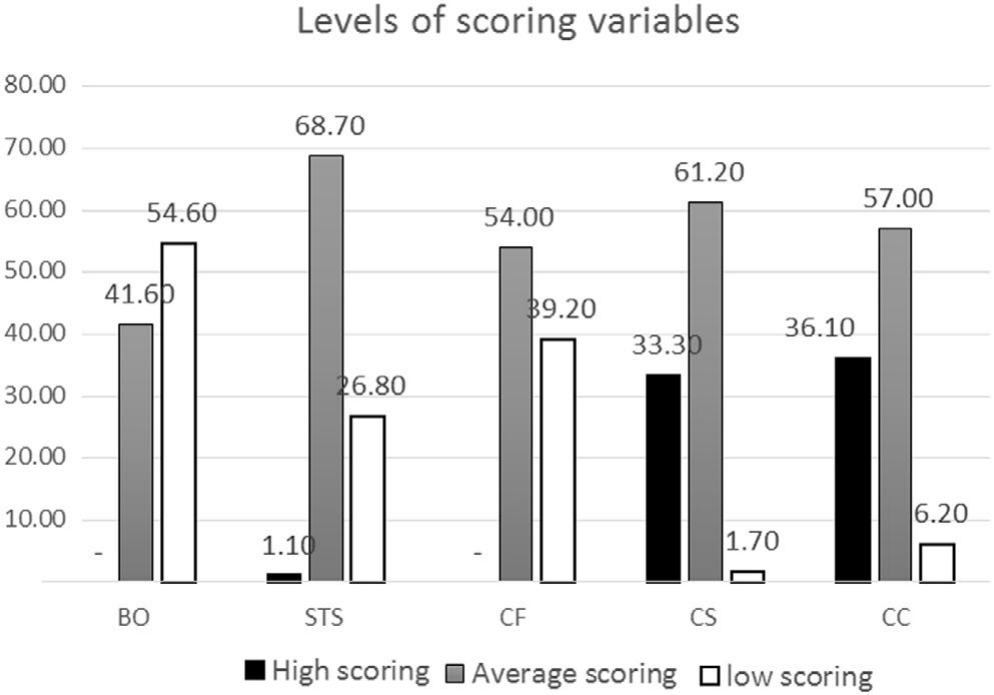
Level of scoring variables in nurse. CS, Compassion Satisfaction; CF, Compassion Fatigue; STS, secondary traumatic stress; BO, Burnout; CC, Clinical Competence

Table 3 shows the participants’ scores on the 7 aspects of clinical competence. As can be seen, the overall clinical competence score was 160.41 (*SD* 28.74). The highest and lowest mean values were related to clinical care (29.35 *SD* 5.82) and professional development (17.14 *SD* 3.67), respectively. Table 4 shows the participants’ scores for the research variables. As shown in Table 4, clinical competence, compassion satisfaction, compassion fatigue, secondary traumatic stress and burnout were assessed based on low, average or high scores. Most participants had average scores for clinical competence (166, 57.0%), compassion satisfaction (178, 61.2%), compassion fatigue (157, 54.0%) and secondary traumatic stress (200, 68.7%) (Figure 1).

**Table 3:** Descriptive analysis of clinical competence (*N* = 291)

Clinical competence (CC)	Mean	*SD*	Min	Max
Clinical care	29.35	5.82	14	40
Leadership	27.25	5.04	15	37
Interpersonal relationships	23.46	4.92	12	32
Ethical/legal performance	23.27	4.488	9	32
Professional development	17.14	3.67	7	24
Coaching/training	17.24	3.75	6	24
Desire for research/critical thinking	22.65	4.70	12	32
Total Clinical competence	160.41	28.74	83	220

Note

Data were presented as mean, *SD*, minimum and maximum for each clinical competence variables.

**Table 4:** Frequency of low, mean, high scores of clinical competence, compassion satisfaction, compassion fatigue, secondary traumatic stress and burnout (*N* = 291)

Variable	*N* (%)	Low	Average	High
Clinical competence	289 (99.3)	18 (6.2)	166 (57.0)	105 (36.1)
Compassion satisfaction (ProQOL)	280 (96.2)	5 (1.7)	178 (61.2)	97 (33.3)
Compassion fatigue (secondary traumatic stress and burnout) (ProQOL)	271 (93.1)	114 (39.2)	157 (54.0)	—
Secondary traumatic stress (ProQOL)	281 (96.6)	78 (26.8)	200 (68.7)	3 (1.1)
Burnout (ProQOL)	280 (96.2)	159 (54.6)	121 (41.6)	—

Note

Data were presented low, mean, high scores for each variable.

Abbreviation: ProQOL, professional quality of life.

### 3.3 Correlational analysis

Table 5 shows Pearson's correlation coefficients between clinical competence and the ProQOL underlying factors. Significant associations were observed between clinical competence and clinical satisfaction (*r* = .334, *p* < .001), compassion fatigue (*r* = −.216, *p* = .001) and secondary traumatic stress (*r* = −.292, *p* < .001). Similarly, significant associations were found between clinical satisfaction and compassion fatigue (*r* = −.459, *p* < .001), burnout (*r* = −.159, *p* = .008) and secondary traumatic stress (*r* = −0.660, *p* < .001). Spearman's correlation coefficient showed a positive association between job satisfaction and compassion satisfaction and negative associations between job satisfaction, compassion fatigue, burnout and secondary traumatic stress (*p* < .05).

**Table 5:** Correlation among the underlying factors of ProQOL and clinical competence (*N* = 291)

Variable	1	2	3	4	5
1. Clinical competence	—				
2. Compassion satisfaction (ProQOL)	0.334** (0.000)	—			
3. Compassion fatigue (secondary traumatic stress and burnout) (ProQOL)	−0.216** (0.005)	−0.459** (0.000)	—		
4. Secondary traumatic stress (ProQOL)	−0.292** (0.000)	−0.660** (0.000)	—	—	
5. Burnout (ProQOL)	−0.095 (0.425)	−0.159** (0.008)	—	0.571** (0.000)	—
6. Job satisfaction^a^	0.102 (0.085)	0.203** (0.001)	−0.273** (0.000)	−0.265** (0.000)	−0.217** (0.000)

Note

Data were presented as Pearson's correlation coefficient.

Abbreviation: ProQOL, professional quality of life.

^a^Spearman correlation.

*p* < .05;

a *p* < .01.**

### 3.4 Regression analysis

Multiple regression models were tested to explore how ProQOL underlying variables and clinical competence can predict compassion satisfaction, secondary traumatic stress and burnout. As shown in Table 6, 65% of the variance of secondary traumatic stress is predicted by compassion satisfaction and burnout (*R* ^2^ = 65%) and the best predictor is compassion satisfaction (*p* < .001). Secondary traumatic stress, clinical competence and burnout predict 53% of the variance of compassion satisfaction (*R* ^2^ = 53%) and the best predictor is secondary traumatic stress (*p* < .001). Compassion satisfaction and secondary traumatic stress predict 40% of the variance of burnout (*R* ^2^ = 40%) and based on β, the best predictor is compassion satisfaction (*p* < .001). Finally, compassion satisfaction predicts 12% of the variances of clinical competence (R^2^ = 12%) and the best predictor is compassion satisfaction (*p* < .001).

**Table 6:** Multiple regression analysis summary for underlying variables of ProQOL and clinical competence (*N* = 291)

Variable	*B*	*SE*	*β*	*t*	*p*	95% CI lower	95% CI upper	*R* ^2^ (%)
Compassion satisfaction								
Constant	46.099	2.410	—	19.113	0.000	41.347	50.852	53
Secondary traumatic stress	−0.971	0.069	−0.818	−14.169	0.000	−1.106	−0.836	
Burnout	0.383	0.065	0.325	5.860	0.000	0.245	0.511	
Clinical competence	0.025	0.010	0.118	2.488	0.014	0.005	0.045	
Secondary traumatic stress								
Constant	29.281	1.633	—	17.934	0.000	26.067	32.495	65
Compassion satisfaction	−0.510	0.031	−0.586	−16.506	0.000	−0.571	−0.449	
Burnout	0.476	0.035	0.480	13.524	0.000	0.407	0.546	
Burnout								
Constant	−5.688	3.166	—	−1.796	0.074	−11.922	0.545	40
Compassion satisfaction	0.345	0.054	0.393	6.405	0.000	0.239	0.451	
Secondary traumatic stress	0.837	0.062	0.830	13.524	0.000	0.715	0.959	
Clinical competence								
Constant	134.987	23.021	—	5.864	0.000	89.629	180.344	12
Compassion satisfaction	1.029	0.413	0.221	2.488	0.014	0.214	1.843	

Note

Data were presented as multiple regression analysis. Only significant results were shown.

Abbreviations: CI, confidence intervals for B; ProQOL, Professional quality of life.

## 4 DISCUSSION

Few studies have directly measured ProQOL, clinical competence and related factors. The results of this study showed that clinical competence was associated with the ProQOL. Specifically, it was found that compassion satisfaction induced by helping patients was associated with clinical competence. This is consistent with a study by Kim et al. that showed nurses with higher compassion satisfaction had a high level of clinical competence in their work and that they were less prone to compassion fatigue and burnout (Kim et al., 2015). According to Soroush et al., (2016) burnout had a negative relationship with nurses’ clinical competence.

Compassion satisfaction and compassionate fatigue act as two modifiers in nursing profession and affect the clinical competence of nurses. Compassion satisfaction in nurses makes them happy with their current situation, creates positive emotions and motivates them to do their jobs more efficiently. On the other hand, compassion fatigue may adversely affect nurses’ performance due to discomfort from observing patients' pain and suffering. Studies have shown that compassion satisfaction is a reward for care and satisfaction with work. It is also related to factors such as the method of care, the functioning of the healthcare system and positive work with colleagues (Yılmaz & Üstün, 2018) all affecting the competence of nurses.

These findings indicated that clinical and nursing managers should implement practical and comprehensive plans to increase nurses’ compassion satisfaction and reduce compassion fatigue as factors affecting clinical competence. In line with the results of this study, O'Callaghan et al. showed that competence in emergency nursing has been a predictor of compassion satisfaction and six descriptive factors related to nurses’ stress, including professional components, play a role in nursing stress (O'Callaghan et al., 2020). Understanding these stressors may help nurses and nursing managers to improve competence levels of nurses. Health organizations need to improve the physical and mental health of nurses by increasing the general well‐being of nurses and implementing training courses such as resilience training and using systems and methods to empower nurses to increase compassion satisfaction and prevent the incidence of cumulative burnout (BO) and compassion fatigue (CF) in the healthcare workforce. As a result, they improve nurses’ performance and job optimization and increase their clinical competence in providing clinical care.

Istomina et al. (2011) showed that nurses’ education, experience, professional development, independence and job satisfaction were factors associated with their competence. The differences in these findings can be attributed to differences in the characteristics of the participants and a greater sample size in the present study compared with similar studies. However, some studies found a significant relationship between clinical competence and demographic factors (Han & Park, 2013; Kang et al., 2011; Meretoja et al., 2015; Slåtten et al., 2010). These results can highlight many factors affecting the competence of nurses, including family support, religious practices and professional work that need further exploration concerning their influence on clinical competence.

The continual increase of clinical competence among nurses is consistent with the rapid changes in the healthcare setting. Nurses’ ProQOL can influence their clinical competence. Kim et al. (2015) showed that technical factors such as competence in providing nursing care and supporting patients increased ProQOL.

The results of this study concerning ProQOL score showed that compassion satisfaction had a protective effect on the compassion fatigue, as highlighted in previous studies (Beaumont et al., 2016; Craigie et al., 2016; Hegney et al., 2015; Heritage et al., 2018; Ray et al., 2013; Sansó et al., 2015). Some studies did not show any negative effects of compassion satisfaction and compassion fatigue (Duarte et al., 2016; Durkin et al., 2016). This is because individuals can regulate their negative states (Lynch et al., 2018). Hegney et al. (2014) found no correlation between compassion satisfaction and secondary traumatic stress. Intervention programmes are necessary in the future to enhance compassion satisfaction and target burnout and secondary traumatic stress. Since secondary traumatic stress involves more acute and disabling symptoms that are too hard to treat (Gentry et al., 2002), we need to set goals and plans to deal with secondary traumatic stress more precisely.

Although the current study found significant correlations between clinical competence, compassion satisfaction, compassion fatigue and burnout, the results of the regression analysis suggested that individuals with increased compassion satisfaction may be particularly resistant to secondary traumatic stress and burnout and have a high level of clinical competence. It means that compassion satisfaction is a key and influential factor that can increase levels of clinical competence and patient care and reduce burnout in nurse.

Even though the present study showed that secondary traumatic stress was associated with compassion satisfaction, compassion fatigue and burnout, it did not show any relationship between secondary traumatic stress and clinical competence. While concepts such as compassion fatigue and secondary traumatic stress are used interchangeably in the literature, compassion fatigue is more common in people who are unable to limit their empathy skills and set their own professional boundaries (Yılmaz & Üstün, 2018).

Meadors et al. (2010) proposed that the terms secondary traumatic stress and compassion fatigue not be used interchangeably. We should examine secondary traumatic stress and compassion fatigue as separate but related constructs and focus on nurses’ awareness of the factors associated with secondary traumatic stress that may help them reduce secondary traumatic stress. Individual‐level protective factors, a strong social support system outside of work and the frequent use of positive coping mechanisms are recommended to reduce secondary traumatic stress (Brady, 2017).

In this study, more than half of the nurses (57.0%) had an average level of competence that was slightly lower than the level reported in Mirlashari et al.’s (2016) study (65.8%). Consistent with the results of the present study, Bahraini et al.’s study showed that most of the nurses reported a good level of competence (Bahreini et al., 2011). This difference can be due to factors such as better educational facilities, more frequent in‐service training programmes and the organization of educational processes.

## 5 LIMITATIONS

There were several limitations in this study. The cross‐sectional nature of this study did not allow the assessment of cause and effect relationship between ProQOL and clinical competence. In addition, the data were collected from two governmental hospitals using a self‐report questionnaire that can restrict the generalizability of the findings. Concerning nurses’ clinical competence at the national and international levels, the socio‐cultural differences should be examined in subsequent studies.

## 6 CONCLUSION

Clinical competence is an essential requirement for the provision of safe and effective care for the patient. This study demonstrated that nurses' compassion satisfaction could increase their clinical competence. It seems that compassion satisfaction can be improved by decreasing the level of secondary traumatic stress and burnout.

## CONFLICT OF INTEREST

No conflict of interest was found for this paper.

## Data Availability

All results of data analysed during this study are included in this published article and its supplementary files. The data set analysed during the current study are available from the corresponding author on reasonable request.
